# Therapeutic Potentials of the Seaweed-Derived Compounds for Alzheimer’s Disease

**DOI:** 10.3390/molecules30224456

**Published:** 2025-11-19

**Authors:** Keanie Ward, Michael H. Cole, Lyn R. Griffiths, Heidi G. Sutherland, Pia Winberg, Barbara J. Meyer, Francesca Fernandez

**Affiliations:** 1 School of Behavioural and Health Sciences, Faculty of Health Sciences, Australian Catholic University, Banyo, QLD 4014, Australia; keanie.ward@myacu.edu.au (K.W.); michael.cole@acu.edu.au (M.H.C.); 2 Healthy Brain and Mind Research Centre, Australian Catholic University, Fitzroy, VIC 3065, Australia; 3 Centre for Genomics and Personalised Health, School of Biomedical Sciences, Queensland University of Technology, 60 Musk Ave, Kelvin Grove, QLD 4059, Australia; lyn.griffiths@qut.edu.au (L.R.G.); heidi.sutherland@qut.edu.au (H.G.S.); 4 Venus Shell Systems Pty Ltd., Huskisson, NSW 2540, Australia; pia@venusshellsystems.com.au; 5 School of Medical, Indigenous and Health Science, University of Wollongong, Wollongong, NSW 2522, Australia; bmeyer@uow.edu.au; 6 Molecular Horizons, University of Wollongong, Wollongong, NSW 2522, Australia

**Keywords:** Alzheimer’s disease, cognitive decline, seaweeds, neuroinflammation, healthy ageing, oxidative stress, neuroprotection

## Abstract

Cognitive decline associated with healthy ageing and pathological conditions is driven by multifactorial processes, including oxidative stress, mitochondrial dysfunction and chronic neuroinflammation. Alzheimer’s Disease (AD), a progressive neurodegenerative disorder affecting cognition and behaviour, is the leading cause of dementia worldwide. Current pharmacological interventions provide modest and transient benefits, targeting limited molecular pathways with safety and cost concerns, underscoring the need for safe, accessible and multi-targeted strategies. This review explores new avenues of therapy with a focus on bioactive compounds derived from brown, red and green seaweeds and their potential to modulate key mechanisms underlying AD. Preclinical and emerging clinical studies demonstrate that phlorotannins, fucoidans, fucoxanthin, lutein, zeaxanthin, ulvan, and astaxanthin exert antioxidant, anti-inflammatory, cholinergic-modulating and neuroprotective effects. Supplementation with seaweed-derived bioactive compounds has been shown to exert molecular and cellular effects that lead to reduced amyloid burden, preservation of synaptic integrity, and enhanced cognitive performance. Collectively, seaweed-derived compounds represent promising candidates for multi-target therapeutic strategies in cognitive decline prevention in the context of AD and healthy brain ageing.

## 1. Introduction

**Figure 1:**
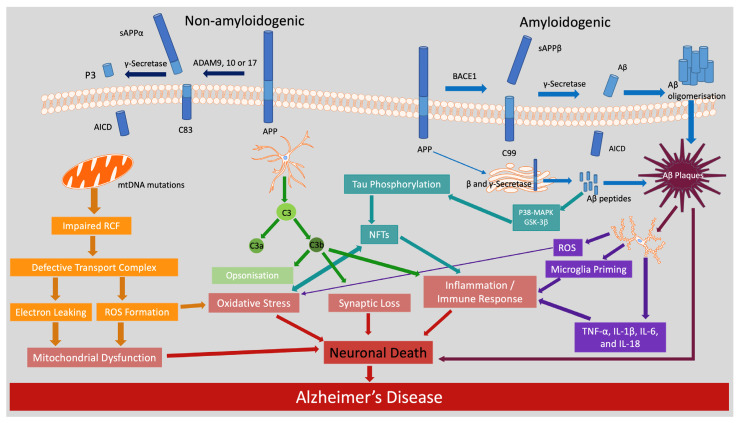
Molecular mechanisms involved in pathophysiology of AD: molecular pathways are colour coded to highlight their contribution to AD pathology. The non-amyloidogenic pathway is dark blue, the amyloidogenic pathway is blue, while mitochondrial dysfunction and oxidative stress are shown in orange, astrocyte and complement activation in green, tau phosphorylation in teal and microglial activation in purple. The colour separation was used to distinguish the overlapping mechanisms and to illustrate how multiple dysregulated pathways converge on neuronal death and AD progression. Abbreviations: ADAM: a disintegrin and metalloproteinase, AICD: APP intracellular domain, APP: amyloid precursor protein, BACE1: β-site APP-cleaving enzyme 1, IL: interleukin, NFTs: neurofibrillary tangles, RCF: respiratory chain function, ROS: reactive oxygen species, sAPPα: soluble APPα, sAPPβ: soluble APPβ, TNF: tumour necrosis factor.

Cognitive decline, whether age-related or indicative of neurodegenerative disorders such as dementia, is an escalating global health concern. As of 2021, it was estimated that 57 million people worldwide were living with dementia, now the seventh leading cause of death among older adults, with Alzheimer’s disease (AD) accounting for 60–70% of cases [1]. While the precise aetiology of AD remains unclear, ongoing research has yielded increasing evidence that disturbance of the biological processes associated with healthy brain ageing contribute to AD. Age is the most prominent risk factor for AD, and it has been suggested that those aged 65 and over have a 1–2% risk, while those aged 85 and over have an 18–38% risk [2,3]. It is crucial to differentiate pathological cognitive decline, observed in AD, from the cognitive changes associated with physiological ageing [4]. AD is characterised by progressive and irreversible cognitive decline in at least one of the main domains, including executive function, language, learning, memory, complex attention, perceptual–motor or social cognition [5]. An earlier literature review has reported that gender differences in incidence rates were most frequently observed in European and US populations, where women consistently showed higher rates than men, particularly at older ages [6]. Although specific risk factors have been identified, the precise neurobiological mechanisms underlying pathological cognitive decline in AD remain only partially characterised. Evidence suggests that several of the cellular and molecular features associated with dementia-related neurodegeneration are present in the healthy ageing brain, albeit to a lesser extent [7]. Key hallmarks of brain ageing, such as oxidative damage, mitochondrial dysfunction, glial cell activation and inflammation, DNA repair deficits and stem cell exhaustion, compromise neural resilience (Figure 1) [8,9,10]. These features are also central in dementia, including AD pathogenesis, underscoring a shared biological continuum between physiological ageing and pathological cognitive decline [7]. AD is characterised by extracellular accumulation of amyloid-β (Aβ) plaques and intracellular neurofibrillary tangles composed of hyperphosphorylated tau, which impair synaptic communication and leads to neuronal death [11]. While a small proportion of cases may be attributed to genetic mutations, most prevailing models, such as the amyloid cascade and cholinergic hypotheses, offer reductionistic explanations that do not account for the disease’s multifactorial and heterogeneous nature [12,13,14,15].

Traditional pharmacological approaches to cognitive decline and impairment in the context of AD primarily target Aβ pathology and cholinergic transmission. Acetylcholinesterase inhibitors (AChEIs), including donepezil, galantamine and rivastigmine, aim to improve acetylcholine (ACh) availability in brain by inhibiting cholinesterase activity. These drugs target cholinergic deficits associated with cortical atrophy, particularly in the frontal and temporal lobes, which are essential for memory [16,17,18]. However, while early studies suggested symptomatic benefits, large randomised controlled trials (RCTs) have shown modest and mixed results [19,20,21]. In a RCT including 769 individuals with mild cognitive impairment (MCI), a possible precursor to AD for some individuals, reported that donepezil administration for 12 months could delay AD progression; however, these initial benefits diminished over the three-year duration of the study [19]. Similarly, the InDDEx trial found that rivastigmine administration for up to several years during the double-blind phase, depending on disease progression or study closure, demonstrated no significant effect on reducing the risk of developing AD or improving cognitive outcomes [20]. Galantamine administration also demonstrated modest cognitive benefits in patients with severe AD when administrated for 26 weeks [21] but was unsuccessful at improving functional outcomes or slowing disease progression and was associated with increased adverse effects in AD patients [21]. Collectively, these findings underscore the ongoing difficulty in developing strategies that reliably mitigate, or slow AD progression. While antipsychotic medications may be used to manage behavioural and psychological symptoms in AD patients, they fail to modify the progression of the disease and have no effect on cognitive decline [22,23]. Additionally, second-generation anti-amyloid monoclonal antibodies (MAB), developed to target Aβ have gained approval as disease-modifying therapies for AD; however, their use remains limited by safety concerns, particularly the risk of amyloid-related imaging abnormalities (ARIAs), which include vasogenic oedema (ARIA-E) and microhaemorrhages (ARIA-H) [24]. This therapy also imposes a significant cost, with annual treatment costs often exceeding $25,000 per patient [25]. Together, the limitation of current pharmacological approaches, including safety risk, high cost and limited efficacy, highlight the need for safer, more accessible and multifaceted therapeutic strategies.

Marine algae, particularly seaweeds, have gained increasing attention as an emerging area of interest due to their rich composition of bioactive compounds with potential neuroprotective properties that may support brain health and potentially mitigate cognitive decline [26]. The therapeutic potential of bioactive compounds derived from seaweeds will be reviewed here focusing on their application in mitigating cognitive decline in normal ageing and AD. Given the growing recognition that AD arises from a convergence of pathological processes, rather than a single causative pathway, current evidence on the key molecular and cellular mechanisms that underpin neurodegeneration will be synthesised. By establishing this pathophysiological framework, we aim to highlight how the properties of seaweed-derived compounds may offer a more integrative and targeted approach to support cognitive health in ageing populations.

## 2. Molecular and Cellular Mechanisms Underlying Cognitive Performance in AD

A major factor contributing to cognitive decline in AD involves the processing of amyloid precursor protein (APP), where the balance between amyloidogenic and non-amyloidogenic pathways influences amyloid deposition, and affects synaptic function, contributing to neurodegeneration.

### 2.1. Amyloid and Non-Amyloidogenic Pathways in AD

The proteolytic processing of APP, a transmembrane glycoprotein predominantly expressed in neurons, follows two primary pathways, amyloidogenic and non-amyloidogenic, yielding different biological outcomes. The amyloidogenic pathway, long regarded as central to AD pathology, involves the initial cleavage of APP by β-site APP-cleaving enzyme 1 (BACE1), generating a membrane-bound C-terminal fragment (C99) and soluble APPβ (sAPPβ), a fragment that lacks the neuroprotective properties of its non-amyloidogenic counterpart (sAPPα) (see Figure 1) [11,14,15]. γ-Secretase subsequently cleaves C99, releasing amyloid-β (Aβ) peptides of varying lengths, most notably the more abundant Aβ1–40 and aggregation-prone Aβ1–42.

Although Aβ40 is the more abundant isoform, Aβ42, characterised by two additional C-terminal residues, exhibits markedly greater hydrophobicity and β-sheet forming propensity, attributing a higher aggregation potential [15,27,28]. This structural distinction promotes rapid nucleation kinetics and thermodynamic stability, allowing Aβ42 to preferentially accumulate in amyloid plaque deposition in the AD brain despite its lower physiological concentration. While Aβ40 can co-aggregate with Aβ42, it appears to exert a modulatory rather than inhibitory role, as mixed Aβ42/Aβ40 oligomers retain comparable neurotoxicity to Aβ42-only species [29,30,31].

*Post-mortem* studies revealed that AD plaques primarily comprise Aβ42 isoforms, and some plaques exclusively contain this isoform of Aβ peptide [29]. An increased Aβ42/Aβ40 ratio has been found to shift the balance toward forming Aβ42-only aggregates, which have been shown to exert the most detrimental effect on neuronal function [30]. Elevated Aβ42 are associated with synaptic plasticity impairments that disrupt calcium homeostasis and initiate oxidative and inflammatory cascades that contribute to synaptic dysfunction and neuronal death; processes associated with cognitive impairment in AD [32,33,34,35]. Furthermore, elevated Aβ42 levels correlated more closely with cognitive decline than total amyloid burden [36]. Thus, the composition and ratio of Aβ isoforms, rather than total concentration, appear to determine neurotoxicity levels and AD progression. While neurons have traditionally been considered the principal cell type responsible for APP expression and Aβ production, recent evidence implicates oligodendrocyte lineage cells (OLGs), including oligodendrocyte precursor cells (OPCs), in this process [37,38]. These glial cells express both β- and α-secretases, supporting their capacity to generate Aβ species and neuroprotective sAPPα [37,39,40]. This finding broadens the neuron-centric paradigm of amyloidogenesis and underscores the importance of investigating multicellular contribution to AD proteopathy.

This duality of APP cleavage is further demonstrated by the non-amyloidogenic pathway, often described as a neuroprotective counterpart to amyloidogenesis, initiated by the cleavage of APP within the Aβ domain by α-secretases, most notably a disintegrin and metalloproteinase domain-containing protein 10 (ADAM10) [41]. This cleavage precludes the formation of Aβ peptides. Instead, it releases sAPPα into the extracellular space and membrane-bound C-terminal fragments (C83). Consequent γ-secretase cleavage of C83 yields a truncated non-toxic fragment (P3 peptide) and the APP intracellular domain (AICD) (See dark blue pathway Figure 1) [42]. In contrast to the sAPPβ, yielded via the amyloidogenesis pathway, sAPPα mediates neurotrophic, synaptogenic and anti-apoptotic effects, supporting neuronal survival and plasticity [43,44,45,46]. Central to these effects is its reaction with the α7 subtype of nicotinic acetylcholine receptors (α7 nAChRs), which are abundantly expressed in the hippocampus and cerebral cortex, regions critical for memory processes and highly susceptible to AD-related degeneration.

Binding of sAPPα to α7 nAChRs facilitates long-term potentiation (LTP) by increasing receptor affinity for endogenous agonists, increasing calcium influx, and activating intracellular signalling cascades, including the phosphatidylinositol 3-kinase/protein kinase B (PI3K/Akt) and mitogen-activated protein kinase/extracellular signal-regulated kinase (MAPK/ERK) pathways [47,48,49]. These molecular mechanisms support synaptic plasticity and neuronal survival [47]. Exogenous administration of sAPPα in aged rat models increased α-amino-3-hydroxy-5-methyl-4-isoxazolepropionic acid (AMPA) receptor surface expression and restored LTP [50]. This synaptic support is mediated through sAPPα’s high-affinity interaction with α7 nAChRs, receptors that are also targeted by neurotoxic Aβ oligomers. This dual ligand affinity highlights the complex functional role of the receptor in AD pathology [50]. While sAPPα binding promotes neuroprotective signalling, Aβ binding induces α7 nAChR de-sensitisation and internalisation, resulting in intracellular Aβ accumulation and calcium dysregulation [51]. These molecular alterations chronically disrupt downstream signalling pathways including cyclic AMP-responsive element-binding protein (CREB) phosphorylation, attenuate AMPA receptor activity, and ultimately contribute to synaptic dysfunction and cognitive decline [48,52]. Thus, APP’s non-amyloidogenic processing averts the formation of neurotoxic Aβ and promotes synaptic resilience through sAPPα-mediated signalling, suggesting this pathway has an important role in supporting neuronal survival and cognitive integrity in the ageing brain [48,52]. While amyloid-β accumulation has long been regarded as an initiating mechanism in AD, evidence suggests its pathological effects alone are insufficient to fully account for the observed neurodegeneration and clinical symptoms. Amyloid deposition appears to act as a catalyst that triggers downstream processes, among which tau pathology has a critical role [53]. The interaction between these two hallmark pathways is increasingly recognised as a point of convergence, whereby amyloid-induced cellular stress promotes tau hyperphosphorylation and aggregation.

### 2.2. Tau Phosphorylation and Neurofibrillary Tangles

Tau is a microtubule-associated protein that has a fundamental role in maintaining the structural integrity and dynamic stability of neuronal axons [54]. By binding to and stabilising microtubules, tau supports essential neuronal processes, such as axonal transport and neurite outgrowth [54]. The affinity of tau for microtubules is finely regulated through post-translational modifications, particularly phosphorylation, which influences both its structural conformation and broader cellular functions [55].

Similar to Aβ, tau exhibits a strong propensity to misfold and form β-sheet-rich aggregates that are associated with neurotoxic effects [56]. This aggregation can occur either through primary nucleation, where tau monomers self-assemble, or secondary nucleation, where existing aggregates accelerate the process [57,58]. Among the various conformations, soluble oligomers and protofibrils are particularly damaging, as they disrupt synaptic signalling and trigger neuronal dysfunction [59]. Importantly, Aβ and tau aggregates can act synergistically: Aβ promotes tau misfolding and phosphorylation (see teal pathway Figure 1), while phosphorylated tau can amplify Aβ-induced mitochondrial damage [59,60,61]. This bidirectional relationship highlights their complementary involvement in the progression of driving AD pathology [62].

Tau hyperphosphorylation in AD results from a complex interaction between dysregulated signalling pathways and alterations in tau isoform expression [63]. In the adult human brain, six distinct tau isoforms are expressed from the MAPT gene on chromosome 17q21 [64]. These arise through alternative splicing of exons 2, 3 and 10, producing variants that differ in both their N-terminal inserts (0N, 1N, or 2N) and the number of microtubule-binding repeats (3R of 4R) [64,65,66]. Isoforms that contain three repeats bind microtubules less tightly than those with four, yet under physiological conditions these two forms are present in roughly equal proportions [64]. In AD, this equilibrium appears to become disturbed, favouring abnormal post-translational modifications, including acetylation, glycosylation, hyperphosphorylation, truncation and conformation changes, which drive self-aggregation and filament assembly [67].

The sequential transformation of tau, from soluble phospho-tau to insoluble fibrillar aggregates forming paired helical filaments, involves converging cellular pathways [68]. The kinases glycogen synthase kinase-3β (GSK3β) and p38- MAPK are commonly implicated in this process (Figure 1) [69,70,71]. Both are abnormally activated in response to oxidative stress, inflammatory cytokines and metabolic dysfunction, further hallmarks of AD [72,73]. Once activated these kinases phosphorylate tau at several disease-associated residues, such as Ser199/202, Tyr 18 and Thr231, reducing its microtubule-binding affinity and contributing to its detachment into the cytosol [74]. Tau hyperphosphorylation decreases its affinity for microtubules and promotes aggregation into NFTs [75]. These aggregates can act as seeds, facilitating further misfolding and prion-like propagation of tau pathology within the brain [76]. The aberrant activation of tau-related kinases such as GSK3β and p38-MAPK not only drives tau hyperphosphorylation but also amplifies oxidative and metabolic stress within neurons [73]. This reciprocal relationship establishes a feedback loop in which oxidative damage further activates these kinases, perpetuating tau aggregation and NFT formation [73]. Consequently, oxidative stress and mitochondrial damage emerge not merely as parallel features of AD pathology, but also as contributing factors.

### 2.3. Oxidative Stress and Mitochondrial Dysfunction

Reactive oxygen species (ROS), a subclass of free radicals, are strongly implicated in the progression of age-related cognitive decline and AD [51]. Oxidative stress arises when ROS production exceeds the brain’s antioxidant capacity, leading to extensive molecular damage [77]. Neurons are particularly susceptible to oxidative injury due to their high metabolic demand and limited regenerative capacity. Although ROS, including hydroxyl radicals (•OH), superoxide anions (O_2_•^−^), hydrogen peroxide (H_2_O_2_) and peroxyl radicals, are considered harmful by-products, several species serve physiological roles as secondary messengers in cell signalling and homeostasis [78,79]. Under conditions of ageing and AD, this physiological balance is disrupted, and excessive ROS accumulation becomes a key contributor of cellular dysfunction and neurodegeneration [80].

Mitochondria are major producers and primary targets of ROS within the cell. Mitochondrial DNA (mtDNA) becomes increasingly damaged with ageing, impairing respiratory chain function and producing defective electron transport complexes that leak electrons and perpetuating ROS formation, establishing a self-reinforcing cycle of mitochondrial dysfunction and oxidative stress (see orange pathway in Figure 1) [81,82]. Excess ROS leads to the oxidation of critical biomolecules, including lipids, proteins, and nucleic acids [83]. Lipid peroxidation damages neuronal membranes and disrupts membrane fluidity, while protein oxidation alters enzymatic activity and impairs neurotransmitter synthesis in the brain [84]. In neurons, this cumulative burden compromises mitochondrial function, synaptic integrity, and cellular viability, thereby contributing to neuronal death [85,86]. ROS can also disrupt dendritic branching, reduce spine density, impair axonal transport, and alter the cytoskeleton, compromising synaptic plasticity and connectivity [84,87,88]. Structural changes are also induced across multiple brain cell types. Elevated ROS can inhibit oligodendrocyte precursor cell maturation, alter branching patterns, and promote demyelination, contributing to the white matter loss reported in ageing and cognitive decline [89,90]. These mechanisms are recognised in the progression of AD and other neurodegenerative conditions. Importantly, the oxidative and structural damage associated with ROS, as well as ROS production can trigger innate immune responses that contribute to sustained microglial activation and the neuroinflammatory cascade observed in AD [91].

### 2.4. Neuroinflammation

Neuroinflammation has a major role in age-related cognitive decline and AD, with the immune cells of the central nervous system serving as key mediators. Astrocytes, the most abundant glial cells in the central nervous system, undergo structural and functional changes with age, contributing to neuroinflammation and cognitive decline [92]. Morphologically, aged astrocytes shift from exhibiting long, slender processes to short, thicker and less ramified extensions; a change observed in humans [93], rodents [94,95], and non-human primates alike [96,97]. Regional differences in astrocyte density have been reported, ranging from reductions in the retina [98] to stability in the hippocampus [99] and increases in the cortex and hypothalamus [100]. Their activation state and molecular signalling appear particularly relevant to age-related neurodegeneration.

One mechanism by which astrocytes contribute to pathological changes in ageing and AD associated with cognitive decline is via the activation of the complement system. Usually dampened in the mature brain, the complement cascade becomes reactivated during ageing and disease, with astrocytes upregulating genes encoding central complement components such as C3 and C4B [101,102]. In particular, C1q and C3 tag synapses for elimination through microglial phagocytosis, a process necessary during development but potentially detrimental when reactivated in the adult brain [103,104]. Astrocytes participate in all three complement pathways, classical, lectin, and alternative, by expressing regulatory proteins and contributing to the cleavage of C3 into C3a and C3b, and facilitating inflammation, and synaptic loss (see green pathway in Figure 1) [104,105,106,107]. While overall neuronal loss is not consistently observed in healthy ageing, loss of synaptic connections is more likely to explain cognitive decline. Memory formation and recall depend on strengthening and reactivating specific synaptic connections. Complement-mediated synaptic pruning by reactive astrocytes may disrupt neuronal networks critical for memory [108]. This leads to the hypothesis that excessive complement activation by aged astrocytes may underlie the synaptic and cognitive deficits recognised in ageing and AD [109,110].

Under physiological conditions, microglia regulate the survival of neurons, differentiation and synaptic remodelling [110]. With ageing, however, these cells undergo progressive morphological and functional changes resembling senescence and dystrophy, rather than classic immune activation [111]. Age-related upregulation of MHC II antigens and altered cytoplasmic morphology are associated with reduced surveillance and host defence capacity, rather than increased pro-inflammatory activation [112]. This suggests that age-related microglial dysfunction may stem from cumulative oxidative stress rather than pathogenic triggers.

Microglial activation, measured by 11C-(R)PK11195 PET, was reported to follow a biphasic trajectory, with early elevations observed in people with mild cognitive impairment compared to controls, followed by longitudinal decline, whereas in AD there is a progressive increase consistent with a shift to a pro-inflammatory phenotype [113,114]. Chronic exposure to Aβ, tau aggregates and neuronal debris is associated with prolonged activation and polarisation towards pro-inflammatory states [115]. Increase in cytokines, including tumour necrosis factor (TNF), TNF-α, prostaglandins and interleukins (IL), IL-1β, IL-6, and IL-18, as well as oxidative stress markers (ROS, nitric oxide (NO)) have been reported in AD and ageing [116]. Collectively, these molecules, while initially serving protective roles, become neurotoxic when overproduced (see purple pathway in Figure 1) [117,118,119]. Notably, microglial-derived TNF and IL-1β promote apoptosis through signalling cascades involving TNFR1, nuclear factor kappa-light-chain-enhance of activated B cells (NF-κB), and caspase-3 pathways, which have been the focus of therapeutic interventions for improving cognitive performance and memory [117,120]. As the disease progresses, microglial phenotypes shift along a spectrum from anti-inflammatory, phagocytic M2-like state to pro-inflammatory M1-like profile, characterised by reduced clearance and heightened cytokine release [117,121]. Although the M1/M2 framework has been criticised as overly simplistic, it remains useful in describing this functional transition.

Additionally, with ageing priming of microglia shows a “dark” or dystrophic morphology and exaggerated inflammatory responses [111,118,122]. These morphologically distinct cells observed near amyloid plaques have been implicated in early tau pathology and may represent a maladaptive stress phenotype rather than a reparative response [122,123]. These phenotypic changes coincide with synaptic loss and tau hyperphosphorylation, hallmarks of cognitive decline in AD [124], suggesting that microglial dysfunction contributes to sustained neuroinflammation and neuronal degeneration. Beyond their immune function, however, microglia are also key modulators of synaptic plasticity [125]. Under homeostatic conditions, microglia enhance neuronal communication through the release of neurotrophic factors, most notably brain-derived neurotrophic factor (BDNF), via PI3K/BDNF signalling pathways [126].

### 2.5. Additional Pathways

BDNF acts as a downstream effector of microglial PI3K, supporting dendritic spine plasticity and synaptic stability in the adult cortex [127]. Reduced expression of BDNF is implicated in the pathogenesis of AD, with mechanistic evidence linking BDNF signalling to neurogenesis and neuronal resilience [128,129,130]. BDNF supports hippocampal memory consolidation and LTP by regulating the expression of genes through the activation of the transcription factors CREB and CREB-binding protein (CBP), which in turn encode proteins involved in synaptic remodelling, stress tolerance, and neuronal survival [128,130]. BDNF signalling becomes compromised by AD-related processes, including Aβ accumulation, oxidative stress and mitochondrial dysfunction [131,132,133]. Reduced BDNF transcription and its downstream TrkB receptor signalling have been linked to disruptions of plasticity-related pathways and accelerating neurodegeneration [134].

*Post-mortem* analyses indicate that higher BDNF expression levels, as measured in regions such as the dorsolateral prefrontal cortex (DLPFC), are associated with significantly slower rates of cognitive decline and a reduced burden of amyloid and tau pathology across multiple cortical regions [135]. In a longitudinal study assessing memory performance, individuals in the highest decile of BDNF expression showed 48.3% slower rates of cognitive decline than those in the lowest decile, suggesting a potential neuroprotective effect of BDNF [135]. A human study provided further evidence for BDNF’s neuroprotective properties, reporting exogenous administration improved cognitive function, restored signalling pathways, and upregulated genes associated with neuroprotection [129]. As a result, BDNF is an increasing focus of intervention research, with ongoing efforts to identify modulators of its expression to mitigate cognitive decline in AD. Collectively, the pathological mechanisms discussed illustrate the complex multifactorial nature of cognitive decline in AD.

Despite the advances in our understanding of the molecular underpinnings of AD, current pharmacological interventions remain limited in their efficacy. Therapies such as AChE inhibitors and anti-amyloid MABs act on target isolated pathological pathways, without adequately addressing the broader network of molecular disturbances that contribute to disease progression [18,24]. This single-target approach is increasingly recognised as insufficient for addressing the heterogeneity and complexity of AD pathology thereby limiting the overall therapeutic impact of current interventions [136]. These limitations have catalysed a shift in therapeutic focus toward compounds capable of modulating multiple pathways simultaneously, particularly those derived from natural sources.

## 3. Therapeutic Potential of Seaweed Compounds in Modulating Brain Ageing Pathways

Therapeutically, natural compounds with pleiotropic actions have gained attention for their ability to target multiple pathological processes in complex brain diseases, such as AD. Several plant-derived compounds, such as curcumin [137], resveratrol [138], epigallocatechin gallate (EGCG) [139], and ginko biloba [140] have shown the capacity to modulate multiple neurodegenerative pathways simultaneously, including oxidative defence, neuroinflammation, Aβ oligomerisation and synaptic plasticity. Amid this growing interest in multi-targeted natural therapeutics, marine macroalgae, commonly referred to as seaweeds, have also gained attention due to their biochemical diversity and long-recognised health benefits [141].

Compared to conventional single-target pharmaceuticals, seaweed-derived compounds may offer broader, multi-targeted approaches to intervention, potentially improving safety and efficacy. Marine algae, particularly edible macroalgae (seaweed) and microalgae, contain a wide range of bioactive properties, with biochemical diversity varying significantly across the main phyla: brown (Phaeophyceae), red (Rhodophyta), and green (Chlorophyta). Each group contains distinct metabolites with unique neuroprotective and therapeutic potentials (see Table 1 and Supplementary Materials for methodology for term search) [142]. A defining feature that sets marine algae apart from terrestrial botanicals is their abundance of sulphated glycans, bioactive polysaccharides absent in land plants [143]. While seaweed also contain antioxidant and anti-inflammatory metabolites common to terrestrial species, sulphated glycans such as fucoidans (brown algae), carrageenans (red algae), and ulvans (green algae) introduce a unique therapeutic dimension [144]. These sulphated polysaccharides possess polyanionic characteristics due to their sulphate group, enabling charge-based interactions that can modulate enzymatic activity, immune regulation, and protein aggregation, pathways that are central to AD pathology [145,146]. Although not every effect can be attributed solely to sulfation, this structural hallmark highlights the therapeutic potential of marine algae as multi-target candidates in cognitive decline and AD. Beyond these mechanistic insights, a growing body of preclinical and clinical evidence indicates that several compounds can enhance memory performance, attenuate oxidative stress in neural tissue, and modulate age-related gene expression associated with neurodegenerative pathways (see Table 1) [147].

### 3.1. Phaeophyceae (Brown Seaweed)

Brown seaweed, varying in pigmentation from yellow to dark brown, represents one of the largest groups of marine macroalgae and accounts for approximately 66.5% of global seaweed consumption [166]. It has attracted scientific interest due to its diverse array of bioactive compounds and its broad range of biological properties. Its unique biochemical composition, comprising up to 70% of tissue mass, containing polysaccharides and other functional constituents, has enabled its application across the nutraceutical, pharmaceutical and cosmetic industries [167]. Brown seaweed is particularly rich in sulphated and non-sulphated polysaccharides, with fucoidan and its derivatives being among the most studied. In addition, they are a primary source of marine carotenoids, which display notable antioxidant, anti-inflammatory, and immunomodulatory properties (see Table 1) [168].

Phlorotannins, such as dieckol, have been shown to exert multifaceted neuroprotective effects by targeting oxidative stress, neuroinflammation, and synaptic dysfunction [148,149]. Dieckol inhibits AChE and restores hippocampal neurotransmitter balance, increasing serotonin and glutamate while reducing GABA and norepinephrine, thereby alleviating memory deficits in ethanol-treated mice [148]. Administration of dieckol has been reported to exert preventive effects by inhibiting oxidative damage and apoptosis through the suppression of caspase activation and mitochondrial dysfunction, while concurrently countering neuroinflammation by inhibiting the NF-κB and MAPK pathways [149,169,170,171]. Dieckol’s antioxidant efficacy is thought to occur via scavenging free radicals (DPPH, ABTS) and reducing intracellular ROS in oxidatively stressed neurons, while activating the Nrf2/HO-1 pathway to bolster endogenous antioxidant defences [169,172,173]. It also mitigates microglia-mediated neurotoxicity by suppressing the release of pro-inflammatory cytokines (TNF-α, IL-1β), responsible for neuronal death (see Figure 1) and by inhibiting NADPH oxidase-driven ROS production in neuronal cell lines [171,174]. In addition, to its antioxidant and anti-inflammatory effects, dieckol has been shown to influence APP processing by enhancing α-secretase (ADAM10) activity while downregulating β- and γ-secretase, promoting the non-amyloidogenic pathway and reducing Aβ accumulation [175]. Dieckol also activates the PI3K/Akt signalling cascade, leading to inhibitory phosphorylation of GSk-3β, a key enzyme in tau phosphorylation and NFT formation (refer to teal pathway Figure 1) [175]. Although there is no direct evidence that dieckol acts directly on tau, preliminary findings indicate potential effects on downstream tau-related pathology [175].

Fucoidans have attracted increasing attention for their neuroprotective and cognition-enhancing properties. Extensive preclinical research demonstrates that fucoidan can ameliorate cognitive impairment across various models of brain dysfunction, including those induced by Aβ infusion, lipopolysaccharide (LPS) exposure and D-galactose administration [151,159,176]. Mechanistically, fucoidans appear to act by reducing oxidative stress and neuroinflammation, restoring mitochondrial function, and modulating key neuroprotective pathways, such as the APN-AMOK-SIRT1 signalling pathway and BDNF expression [151,159]. Emerging evidence also suggests that fucoidans may also interact with molecular processes linked to tau pathology. Preliminary *in vitro* findings indicate that certain sulphated fucoidan fractions, such as galactofucans and heteropolysaccharides, may compete with cell-surface heparan sulphate for tau bindings sites, potentially reducing tau attachment and internalisation [177]. By interfering with this receptor-mediated uptake, fucoidan may theoretically limit the cell-to-cell propagation of tau aggregates and the subsequent formation of NFTs [177].

In animal studies, fucoidan improved performance in learning and memory tasks and ameliorated biochemical markers of neuronal damage [152]. Specifically, it has been shown to restore ACh levels and enhance biochemical markers of neuronal health, including reducing markers of apoptosis in the hippocampus [152]. These effects are attributed to decreased lipid peroxidation, inhibition of AChE activity, and suppression of caspase-mediated apoptosis, collectively indicating a broad mechanism of action involving oxidative stress reduction, mitochondrial protection, and anti-apoptotic effects [26]. Additionally, *Sargassum muticum*, including fucoidan compounds, has been shown to protect cells from oxidative stress induced by neurotoxins, such as 6-hydroxydopamine (6-OHDA) in *in vitro* studies [178]. Emerging evidence also suggests that fucoidan may exert its neuroprotective effects through epigenetic mechanisms. In a human clinical study, a single oral dose of *Undaria pinnatifida* fucoidan significantly altered the expression of 53 circulating microRNAs and modulated 31 cellular pathways, several of which are associated with neurotrophic signalling, including BDNF, MAPK, EGFR, and insulin receptor pathways [179].

Another brown seaweed-derived compound phloroglucinol, a polyphenolic compound, has demonstrated neuroprotective activity in both *in vitro* and *in vivo* models of AD. In studies using the 5XFAD transgenic mouse model, which recapitulates key features of AD, oral administration of phloroglucinol reduced Aβ plaque burden and lowered protein levels of BACE1, a key enzyme in Aβ production (see blue pathway Figure 1) [156,157]. Additionally, it markedly decreased oxidative stress markers, including 4-hydroxynonenal (4-HNE) and suppressed glial activation as evident by reduced levels of GFAP, an astrocyte marker, and Iba-1, a microglia marker [156,157]. These molecular changes were accompanied by restoration of dendritic spine density in the hippocampus and improvements in spatial learning and memory in 5XFAD mice when compared to controls [156,157]. Phloroglucinol exerts its effects through potent antioxidant activity, scavenging ROS and enhancing endogenous antioxidant enzyme levels, likely via upregulation of the Nrf2 pathway [157,180]. These findings suggest that phloroglucinol may mitigate key neuropathological features of AD and improve cognitive function.

Seaweed-derived carotenoids, such as fucoxanthin, have been explored in both preclinical and clinical studies. In D-galactose-induced ageing mouse models, fucoxanthin supplementation attenuated cognitive deficits by reducing lipid peroxidation and lowering pro-inflammatory cytokines, specifically TNF-α and IL-6 [152]. In addition to its effects on oxidative and inflammatory pathways, oral administration of fucoxanthin has also been shown to reduce tau accumulation in the brains of treated mice [181]. However, it is speculated that this is likely secondary to the antioxidant and anti-inflammatory properties rather than a direct effect on tau [181]. Clinical trials in older adults with self-perceived cognitive decline reinforce these findings. For example, in a 12-week randomised, placebo-controlled trial, adults (*n* = 22) receiving 8.8 mg/day fucoxanthin supplementation demonstrated significant within-group improvements in delayed recall (+1.2 words), executive function (Stroop reaction time −42 ms), attention (digital vigilance error −13%), and vigilance (choice reaction time −46 ms), although most between-group differences compared with placebo (*n* = 21) were not statistically significant [153]. Furthermore, six months of daily supplementation with a lower dose, 4.4 mg/day, resulted in significant gains in both immediate and delayed word recall [182]; further emphasising enhancements in both working and episodic memory in an intervention study with an elderly cohort.

Brown seaweed-derived phytosterols, particularly fucosterol and 24(S)-saringosterol from *Himanthalia elongata* and *Sargassum fusiforme*, have been shown to activate the liver X receptor beta (LXRβ) selectively, a nuclear receptor critical for cholesterol homeostasis and amyloid clearance in the brain [183,184,185]. Importantly activation of LXRβ is achieved with minimal activation of LXRα, thereby reducing the risk of hepatic side effects that are commonly associated with non-selective LXR agonists [183,184,185]. *In vitro* studies confirm that extracts from these seaweeds, especially those from *Sargassum fusiforme*, robustly activate LXRβ, but not LXRα, with this activation selectively attributed to 24(S)-saringosterol [183]. Dietary supplementation of *Sargassum fusiforme* for 12 weeks has led to an accumulation of 24(S)-saringosterol in the brain, upregulating LXR target genes, decreasing astrocyte activation and improving performance in object and spatial memory in APPswePS1ΔE9 mouse model (see Table 1) [154]. Collectively these studies suggest that brown seaweed phytosterols represent a potential therapeutic strategy without the adverse hepatic effects seen with pan-LXR agonists.

The range of neuroprotective actions attributed to brown seaweed bioactive compounds illustrates their potential therapeutic relevance across multiple molecular targets involved in AD pathology. By addressing several interconnected mechanisms, these compounds offer the potential for a multifaceted intervention strategy that is not achieved by conventional single-target therapies. Given this emerging evidence, research has begun to explore red seaweeds, which offer a distinct phytochemical composition in comparison to brown seaweed.

### 3.2. Rhodophyta (Red Seaweed)

Red seaweeds, or red algae, are a diverse group of marine macroalgae recognised for their rich bioactive compounds and pigments, including carotenoids, chlorophyll a and d, phycoerythrin, phycocyanin, and allophycocyanin [186]. These pigments not only impart the characteristic red hue, but also contribute to antioxidant capacity, supporting their relevance in nutritional and therapeutic contexts [187]. A hallmark of red seaweeds is their abundant cell wall polysaccharides, particularly agar, carrageenan and sulphated galactan, that exhibit antioxidant, anti-inflammatory and neuroprotective properties [188].

Sulphated polysaccharides derived from red seaweeds, such as those isolated from *Gelidium pristoides*, have been shown to disrupt Aβ fibril formation in *in vitro* studies, indicating a direct anti-aggregation effect that targets a pathological feature of AD (see Figure 1) [189]. In addition to this anti-amyloidogenic action, enzymatically digested κ-carrageenan oligosaccharides (KOS), another class of red seaweed polysaccharides, have been found to attenuate neuroinflammation by inhibiting microglial hyperactivation and reducing the expression of pro-inflammatory cytokines, specifically IL-1β, TNF-α, and IL-6, in *in vivo* and *in vitro* AD models [26]. KOS, significantly attenuated hydrogen peroxide-induced cytotoxicity in PC12 cells, reducing intracellular ROS and preserving mitochondrial membrane potential, indicating protection against oxidative-stress-induced apoptosis [190]. Derivatives of KOS, pentasaccharides, were found to protect neurons from Aβ-induced apoptosis by suppressing the overactivation of the c-Jun N-terminal kinase (JNK) signalling pathway, a mediator of Aβ neurotoxicity [191].

Lutein and zeaxanthin, xanthophyll carotenoids found in both red and green algae, have attracted increasing research interest in the context of AD, compared to other red algae bioactive compounds. Supplementation with these carotenoids in pre-clinical studies has been reported to mitigate memory deficits, reducing Aβ deposition and neuroinflammation [192,193,194]. Lutein and zeaxanthin are thought to exert their effects through multiple pathways, involved in oxidative stress, AChE activity, and downregulation of pro-inflammatory cytokines such as TNF-α and IL-1β [192]. Furthermore, neuronal health is supported by increasing neurotrophic factors such as BDNF and ciliary neurotrophic factor (CTNF), and by maintaining glucose homeostasis in the brain [192]. Epidemiological studies and clinical trials found that a higher dietary intake and serum levels of lutein and zeaxanthin were associated with improved episodic memory, visual learning and a reduced risk of cognitive decline and dementia [160,193,194,195].

Together, the bioactive profile of red seaweeds appears to confer neuroprotective effects through multiple mechanisms. These effects, while overlapping with those of other phyla, arise from compounds unique to red algae, underscoring the importance of phylum-specific profiles and research in targeting multiple facets of AD pathology. Although red and green seaweeds share broad classes of bioactive compounds, such as polysaccharides, their specific molecular constituents differ considerably.

### 3.3. Chlorophyta (Green Seaweed)

Green algae are primarily distinguished by their high content of chlorophyll, a green, lipid soluble pigment that is ubiquitous among plants, algae and cyanobacteria [196]. Beyond the chlorophyll content, green seaweeds, like the other phyla contain a rich reservoir of bioactive compounds with potential therapeutic relevance for AD. Although green seaweeds exhibit a lower total polysaccharide content relevant to brown and red seaweeds, they are particularly enriched in ulvan, a sulphated polysaccharide [197].

*In vitro* research has shown that ulvan-rich extract, derived from *Ulva lactuca*, exhibits notable antioxidant and anticholinesterase activity, restoring cell viability and inhibiting caspase-3 activation in BPA-challenged SH-SY5Y neuroblastoma cells, findings that directly address oxidative stress and apoptosis [164]. Beyond cell studies, ulvan’s antioxidant effects have been demonstrated *in vivo* to increase superoxide dismutase and glutathione peroxidase activity, reducing lipid peroxidation and decreasing cytokines such as TNF-α and IL-6 in pre-clinical studies [198]. Ulvan extracts were found to lower markers of biomolecular damage, including protein carbonylation and DNA oxidative lesions, and protected against apoptosis by downregulating caspace-3 expression in animal model [198]. Ulvan anti-inflammatory and immunomodulatory properties have been confirmed in additional studies, which suggests that its biological activity depends on factors such as molecular weight, sulphate content and carbohydrate composition [199]. Ulvan broader therapeutic potential also supports its modulation of immune response [200].

Astaxanthin, a xanthophyll carotenoid belonging to the terpene family, is widely distributed in marine organisms. The microalgae *Haematococcus pluvialis,* can accumulate astaxanthin up to 3.8% of its dry weight, with evidence suggesting potential neuroprotective effects [201]. Preclinical studies in AD have shown that astaxanthin supplementation results in the amelioration of memory deficits, reduction in Aβ deposition in the hippocampus and cortex, decreased tau phosphorylation and parvalbumin-positive neuron density in the hippocampus of AD model mice compared to control mice [202,203,204]. In these pre-clinical studies, enhanced antioxidant capacity and modulation of microglia and pro-inflammatory marker expression were reported in the hippocampus of AD mice models [205,206]. Astaxanthin appears to exert its neuroprotective actions by attenuating oxidative stress, reducing neuronal apoptosis, and suppressing neuroinflammatory processes via SIRT1/PGC-1α signalling pathway [179]. Furthermore, upregulation of anti-apoptotic proteins (Bcl-2), and downregulation of pro-apoptotic proteins (like Bax), were also reported in these AD mice models, inhibiting potentially neuronal senescence and apoptosis induced by Aβ toxicity [206].

Clinical evidence regarding the effects of astaxanthin on cognitive function in humans remains limited but is gradually emerging, with several randomised, double-blind, placebo-controlled trials offering preliminary insights into its potential therapeutic benefits as well as its limitations (see Table 1). Supplementation of astaxanthin in a human cohort supports preclinical findings as phospholipid hydroperoxide levels in erythrocytes significantly decreased, indicating a reduction in lipid peroxidation and systemic oxidative damage [162]. In a study by Katagiri et al. (2012), supplementation with astaxanthin-rich *H. pluvialis* extract led to significant improvements in cognitive performance, particularly psychomotor and processing speeds in middle-aged and older adults (see Table 1) [161]. Similarly, a systematic review of clinical trials found that astaxanthin supplementation was associated with subjective and objective improvements in cognitive function among older adults, although not all studies reached statistical significance [207]. Subgroup analyses have indicated that younger participants, aged 45–54 years, may experience more pronounced cognitive benefits, especially in memory tasks, in comparison to older cohorts [208].

These findings underscore the potential of green seaweed-derived compounds in mitigating the neurodegenerative processes associated with AD. Ulvan’s potent antioxidant, anti-apoptotic, and anti-inflammatory activity, alongside astaxanthin’s capacity to modulate mitochondrial function, position green algae as another promising source of neuroprotective agents. Although green seaweeds possess a comparatively narrower diversity of characterised bioactive compounds than its brown and red counterparts, emerging preclinical and early clinical evidence indicate their continued investigation within a multifaceted, integrative framework for AD intervention.

## 4. Discussion

Evaluation of the therapeutic potential of brown, red and green seaweeds provides a strong rationale for their consideration in strategies addressing neurodegeneration and cognitive decline in AD. Bioactive compounds from brown, red and green algae share convergent mechanisms that influence key molecular processes involved in AD. Across all phyla, antioxidant activity represents a recurrent characteristic; compounds such as dieckol and fucoxanthin (brown), carrageenans and xanthophylls like lutein and zeaxanthin (red), and ulvan (green) have been reported to reduce intracellular ROS, enhance mitochondrial stability, and activate the Nrf2/HO-1 antioxidant defence pathway [164,172,192]. Anti-inflammatory effects are also frequently observed, with these compounds downregulating NF-κB and MAPK signalling, suppressing microglial overactivation, and reducing pro-inflammatory cytokine expression [171,198,205]. Additionally, seaweed compounds have been reported to modulate neurotrophic and cholinergic pathways by upregulating BDNF and CTNF, inhibiting AChE activity, and reducing caspase-3-mediated apoptosis, mechanisms supporting synaptic plasticity, neuronal survival and cognitive performance [151,194,198]. Together, these pleiotropic actions suggest the therapeutic potential of seaweed in addressing the multifactorial pathology of AD.

**Figure 2:**
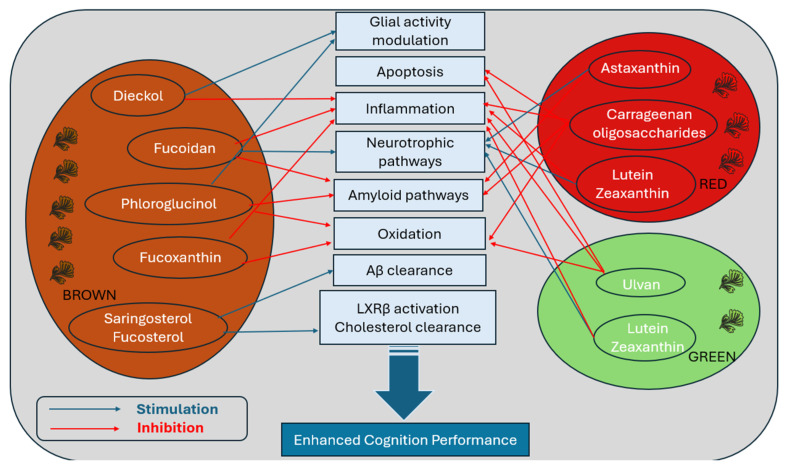
Summary of seaweed derived compounds from brown, red and green algae on major AD molecular pathways. The red arrow represents an inhibition effect while the blue arrow illustrates stimulation. Abbreviations: LXRβ: liver X receptor beta; Aβ: amyloid beta.

Importantly, these molecular effects are paralleled by reported improvements in cognitive function (see Figure 2). Across both preclinical and clinical models, seaweed-derived compounds have associated with improvements in memory, learning, and attention. In animal models of AD, brown seaweed constituents such as dieckol, fucoidan, fucoxanthin, and phloroglucinol improved memory performance [148,151,161]. Similar effects were observed with LXR-activating compounds like saringosterol, which improved spatial and object memory in transgenic AD mice [154,184]. While human trials remain limited, early evidence suggests cognitive benefits in domains such as memory and processing speed following supplementation with astaxanthin, lutein and zeaxanthin, and fucoxanthin [153,160,161]. This evidence supports the potential of specific seaweed-derived compounds in addressing cognitive decline. While clinical trials remain limited, these early findings suggest cognitive benefits in humans, supporting the potential role of seaweed compounds as multi-target interventions for cognitive impairment associated with ageing and AD. Notably, related effects are not exclusive to age-related and pathological decline, as macular xanthophylls, a green seaweed-derivative demonstrated efficacy in improving cognitive outcomes in young adults [163].

Available studies to date indicate favourable safety and tolerability. Clinical trials involving *Ulva lactuca* and brown seaweed extracts have reported no notable adverse effects over several weeks to months of supplementation [209,210]. Given the convergence of mechanisms across different seaweed species and bioactive compounds, the translational potential of this research appears encouraging. These compounds which target multiple hallmarks of cognitive decline and AD, position them as potential candidates for future adjunctive or preventative strategies.

Despite promising early findings, current clinical research remains constrained by methodological limitations that restrict the generalisability and strength of conclusions. For example, within green seaweed-derived compounds, Katagiri et al. (2012) conducted a 12-week randomised, double-blind, placebo-controlled trial examining astaxanthin supplementation (6 or 12 mg/day) in 96 middle-aged and older adults [161]. While the results indicated cognitive benefits, the relatively low dosage and short intervention period limit conclusions regarding long-term efficacy or optimal dosing.

Similarly, Lopresti et al. (2022) evaluated the effects of lutein (10 mg/day) and zeaxanthin (2 mg/day), red seaweed-derived compounds, over six months in 51 adults; however, the modest sample-size, limited follow-up and inclusion of a generally healthy middle-aged population may limit the applicability of these findings to older adults experiencing cognitive decline or at risk of AD [160]. While 10 mg of lutein and 2 mg of zeaxanthin fall within the range commonly used in seaweed-related nutritional neuroscience studies, evidence suggests these doses may be lower than those producing stronger cognitive effects from other studies, as a dose equivalent to 10 g of dried brown seaweed has shown greater efficacy in enhancing acute cognitive performance in humans [210]. It also remains unclear whether longer exposure, higher doses, or trials in more clinically relevant populations would yield more robust or translatable outcomes. Potential adverse effects may remain undetected in short-term studies involving healthy individuals but may occur at a later stage. Furthermore, red seaweed compounds, like sulphated polysaccharides, lutein and zeaxanthin, remain understudied in human trials, with most evidence deriving from preclinical animal models. The long-term safety and efficacy of high-dose supplementation in humans remains limited.

In a brown seaweed compounds focused study, Yoo et al. (2024) administered fucoxanthin (8.8 mg/day) to a population of adults between 55 and 75 years with subjective cognitive decline, but this study was limited by the cohort age and size (*n* = 43), brief follow-up duration (12 weeks) and low dosage [153]. In contrast to the modest clinical studies, preclinical studies in animal models often report greater improvements at higher per-body-weight doses, such as dieckol (20 mg/kg/day) [148], fucoidan (100 mg/kg/day) [151], fucoxanthin (50 mg/kg/day) and phloroglucinol [156], and saringosterol (10 mg/kg/day) [154]. However, it is recognised that these doses often exceed those that would be considered safe or practical in humans [211].

When integrating current findings across seaweed phyla, evident disparities emerge in the scope, quality, and translational progress of the evidence base. Brown seaweeds remain the most extensively studied, with a growing body of both preclinical and early clinical data supporting the potential for cognitive benefits via antioxidant, anti-inflammatory, and neurotrophic mechanisms. However, existing human trials are often constrained by small sample sizes, brief intervention durations, and subtherapeutic dosing, highlighting the need for larger, well-powered studies targeting AD-relevant populations. In contrast, red seaweed, despite being rich in bioactive xanthophylls and sulphated polysaccharides, remains predominantly explored in preclinical models, with limited human data. Green seaweeds similarly show promising neuroprotective potential *in vitro* and *in vivo*, particularly through ulvan and astaxanthin-mediated antioxidant and anti-apoptotic effects. Yet, clinical evidence remains minimal.

Seaweed-derived bioactive compounds present several physicochemical and pharmacokinetic limitations that challenge their translation into AD therapeutic agents. Brown seaweed-derived compounds, such as phlorotannins and fucoxanthin, have shown poor solubility in aqueous solution (such as plasma) and chemical instability under physiological pH and temperature, leading to their degradation in preclinical studies [212]. Oral administration of phlorotannins compared to IV administration in rats demonstrated the extensive first-pass metabolism in liver, resulting in a low systemic bioavailability [212]. In contrast, sulphated polysaccharides such as fucoidan and laminarin are more water-soluble and their absorption varies according to their molecular weight (differing with sulfation pattern) [213]. High digestion and absorption efficiency was reported with the lower molecular weight of fucoidan [214] while the higher molecular weight fucoidan is often more viscous than laminarin, affecting its absorption and permeability to brain blood barrier (BBB) [215,216].

These physicochemical and pharmacokinetic constraints may limit the concentrations of ingested seaweed-derived compounds in the systemic circulation and, consequently, in the brain. Improving the bioavailability of seaweed-derived compounds remains a key challenge, however novel delivery strategies such as nanoparticle encapsulation, phytosome delivery, and conjugation with carrier molecules have recently emerged and show potential for improving bioavailability [215,217,218].

Nanoencapsulation of fucoxanthin increased its bioavailability when compared with administration of the same non-encapsulated seaweed-derived compound in animal model (encapsulation efficiency of fucoxanthin over 78%), which may open new avenues for effective delivery of seaweed-derived compounds in the human body [217].

Similarly, fucoidan’s biological versatility and its gel-forming properties for nanogel preparation, alongside of red seaweed polysaccharides such as carrageenan, offer biocompatible and sustained matrices for controlled drug release, potentially enhancing intestinal or BBB transport [218]. Phytosomes, phospholipid-based nanocarriers that mimic cell membranes, can protect seaweed-derived compounds from degradation form digestive system, improve their bioavailability and increase their transport via BBB [219]. Conjugation of seaweed compounds with negatively charged carbon dot molecules such as fucoidan-derived carbon dots, may enhance BBB transport through their small size and negatively charged surface [220]. With the presence of sulphate and hydroxyl groups derived from fucoidan further facilitating uptake and conferring neuroprotective effects in neuronal cells and animal models of Parkinson disease [220].

These emerging delivery systems, which improve biocompatibility and stability, may enhance the therapeutic potential of seaweed compounds, although further validation in human trials is required to optimise compound efficacy and ensure safety.

To advance clinical translation, future research should prioritise phylum-specific investigations, dose–response relationships, and longer intervention durations in at-risk older adults. Carefully designed pilot trials are warranted to assess the safety, feasibility and preliminary efficacy, followed by larger rigorously controlled studies, to evaluate long-term outcomes and identify any delayed or dose-dependent adverse effects that may not be apparent in short-term trials involving healthy individuals.

## 5. Conclusions

Existing research proposes seaweed-derived bioactive compounds as potential multi-targeted approaches to target the complex pathology of AD, addressing interconnected mechanisms such as oxidative stress, neuroinflammation, amyloid-β aggregation and synaptic dysfunction. Preclinical studies have reported some neuroprotective effects across brown, red and green algae, with early clinical trials indicating safety and potential cognitive benefits. However, at this point translation to clinical settings is limited by small sample sizes, low dosages, short intervention periods and under-representation of at-risk populations. Advancement in this field will benefit from well-designed and adequately powered human studies that evaluate long-term efficacy, optimal dosing, and potential synergistic effects of combined bioactive compounds. Nonetheless, emerging evidence indicates that integrating these natural compounds into prevention or adjunctive treatment strategies, offers the potential to improve cognitive outcomes in ageing populations and those at risk of AD. Given the global ageing population, the exploration of natural compounds as cognitive support interventions is increasingly relevant for t both healthy individuals and people living with pathological conditions such as AD.

## Figures and Tables

**Table 1:** Key Bioactive Compounds in Brown, Red, and Green Seaweeds Relevant to Cognition and AD pathophysiology.

Compound	Chemical Structure	Algal Source	Mechanism	Tested Model	Main Findings	Reference
Dieckol	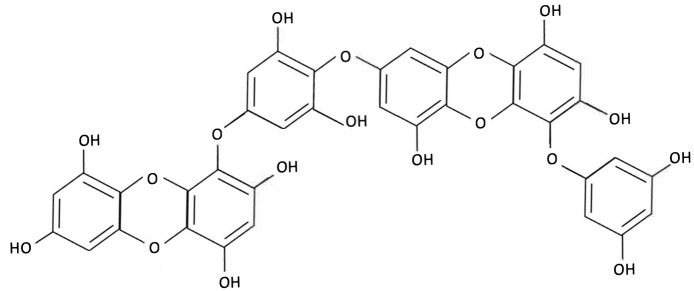	Ecklonia cava, Brown Seaweed	AChE inhibition, Neurotransmitter modulation	Male ICR mice received oral dieckol (1 or 10 mg/kg/day) for 7 days. Cognitive performance was assessed via passive avoidance test; brain neurotransmitter levels and AChE activity were measured.	Significantly improved memory in ethanol-treated mice, restored hippocampal 5-HT and glutamate levels, reduced elevated GABA and norepinephrine levels, increased brain ACh and inhibited AChE activity (IC_50_ ≈ 17.5 µM).	[148]
Dieckol	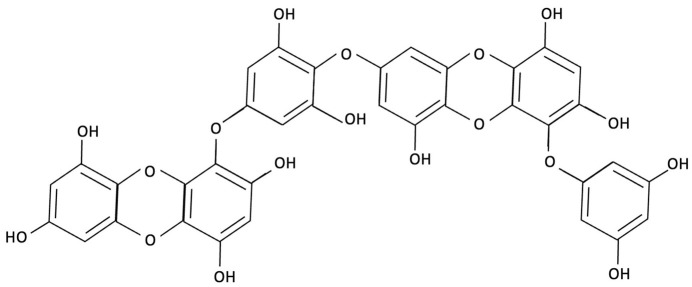	Ecklonia cava, Brown Seaweed	Antioxidant, Anti-inflammatory, Antiapoptotic, NF-κB and MAPK signalling	*In vitro*, using PC12 cells treated with Aβ_(25–35)_ simulating AD-like neurotoxicity. Phlorotannins were administered at varying concentrations 1 h before Aβ.	Dieckol significantly restored PC12 cell viability, reduced Aβ25–35–induced oxidative stress, inflammation, and apoptosis, and modulated NF-κB and MAPK signalling pathways.	[149]
Diphlorethohydroxycarmalol (DPHC)	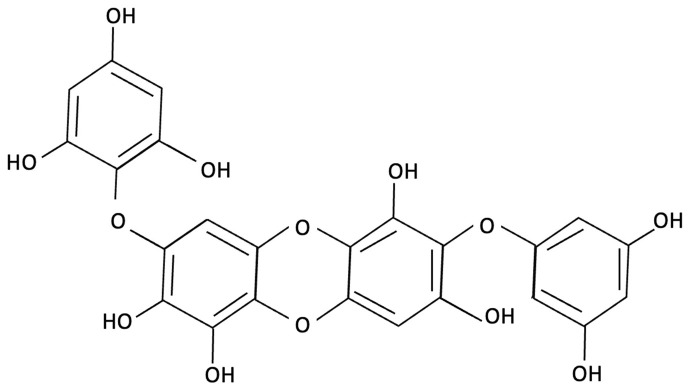	Ishige okamurae, Brown Algae	Anti-inflammatory, Neuroprotective	AD-like cognitive impairment induced in male C57BL/6 mice via ICV injection of Aβ25–35. *In vitro*, PC12 neuronal cells used for further investigation of molecular mechanisms related to oxidative stress, apoptosis and MAPK pathway activation.	Significantly attenuated Aβ_(25–35)_–induced cognitive impairment, improved maze test performance. IOE reduced neuronal apoptosis, cleaved caspase-3 and PARP, suppressed neuroinflammation (iNOS, COX-2), and decreased ROS overproduction. Both *in vivo* and PC12 cell models, IOE reversed abnormal phosphorylation of ERK, p38 MAPK, and JNK.	[150]
Fucoidan	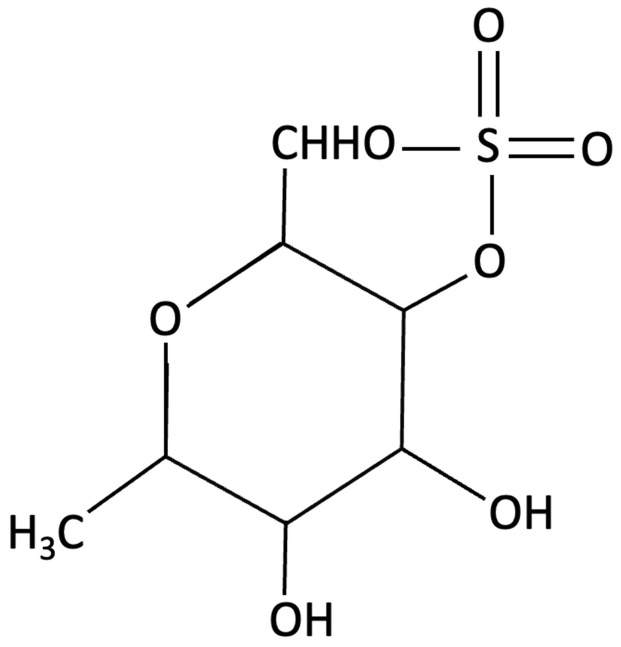	Fucus vesiculosi, Brown Algae	Neuroprotective	Male C57BL/6 mice treated with LPS and oral fucoidan (10 mg/kg) daily for three weeks.	Attenuated LPS-induced cognitive impairment by reducing neuroinflammation, oxidative stress, AChE activity. Enhancing BDNF expression and neurogenesis.	[151]
Fucoxanthin	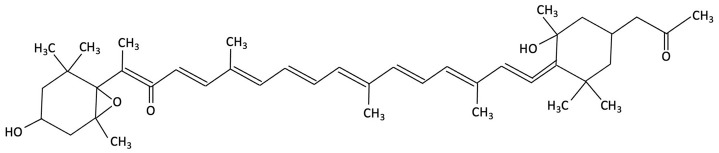	Phaeodactylum tricornutum, Brown Algae	Antioxidant, Anti-inflammatory	D-galactose-induced ageing mouse model using male Swiss mice (*n* = 72), treated with PT extract for 79 days. Cognitive performance assessed via Y-maze, Morris Water Maze, and Passive Avoidance tests.	Significantly reversed induced cognitive impairment in Y-maze, Morris Water Maze and Passive Avoidance tests, reduced hippocampal lipid peroxidation, and decreased elevated TNF-α and IL-6 levels in brain and plasma, particularly at higher doses.	[152]
Fucoxanthin	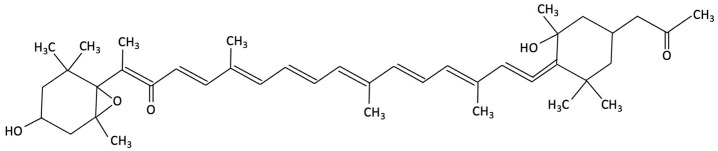	Phaeodactylum tricornutum, Brown Algae	Antioxidant, Anti-inflammatory	12-week double-blind, randomised, placebo-controlled clinical trial involving older adults (55–75 years) with age-associated memory impairment. Assessing 8.8 mg/day supplementation on cognitive performance and inflammatory biomarkers	Improved working and episodic memory, attention, vigilance and executive function. Inflammatory cytokines showed minimal changes, a slight increase in IL-1β and stable TNF-α and IL-6 levels.	[153]
Fucosterol and Saringosterol	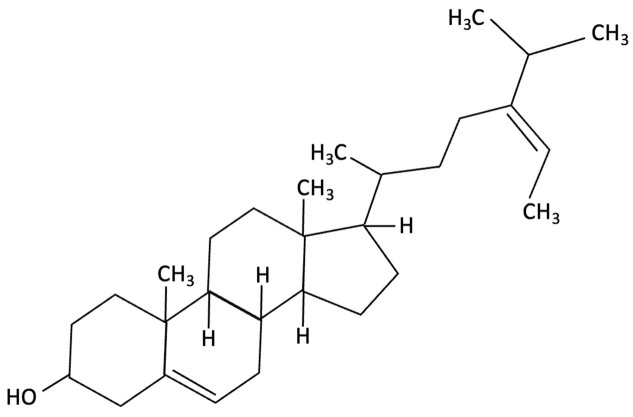 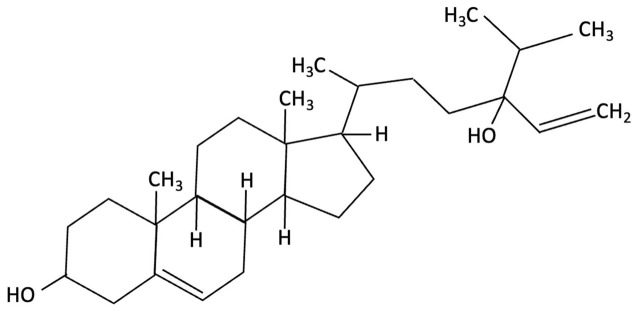	Himanthalia elongate, Sargassum fusiforme, Brown Seaweed	Anti-inflammatory	*In vitro* testing using LXR luciferase reporter assays and *in vivo* via 12-week dietary supplementation in APPswePS1ΔE9 mice.	Significantly prevented cognitive decline in APPswePS1ΔE9 mice across object, spatial, and working memory tasks. Both *H. elongata* and *S. fusiforme* extracts reduced cortical GFAP expression, suggesting attenuation of astrocyte activation.	[154]
Lycopene	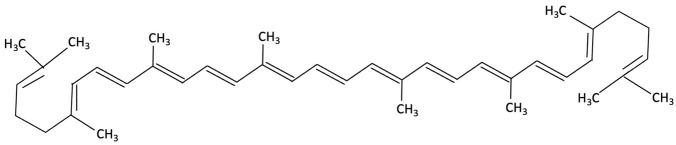	Dictyota spiralis, Brown Seaweed	Neuroprotective	Male C57Bl/6J mice (3-month) administered lycopene-supplemented diet (0.3% *w*/*w*) for five weeks. 9 days of LPS induction of neuroinflammation.	Alleviated LPS-induced amyloidogenesis and memory loss, through inhibited microglial activation, reduced inflammatory mediators and enhances antioxidant enzymes, partly via modulating MAPK, NF-κB, PI3K/Akt, and Keap1/Nrf2 pathways.	[155]
Phloroglucinol	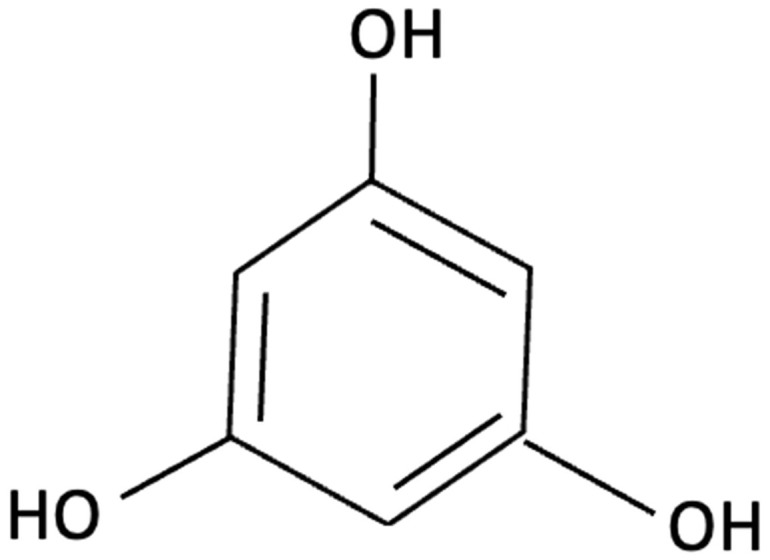	Ecklonia cava, family Laminariaceae, Brown Seaweed	Antioxidant, Anti-inflammatory, Aβ metabolism	5XFAD transgenic mouse model of Alzheimer’s disease used to assess the neuroprotective effects of orally administered phloroglucinol (100 mg/kg/day) over 2 months.	Significantly improved cognitive performance in 5XFAD mice (T-maze and Y-maze), reduced Aβ protein levels and plaque burden, lowered oxidative stress (↓4-HNE), and suppressed glial activation (↓GFAP, ↓Iba-1). Additionally, decreased pro-inflammatory cytokines (TNF-α, IL-6), reduced BACE1 expression, and restored dendritic spine density and mature spine morphology in the hippocampus.	[156]
Phloroglucinol	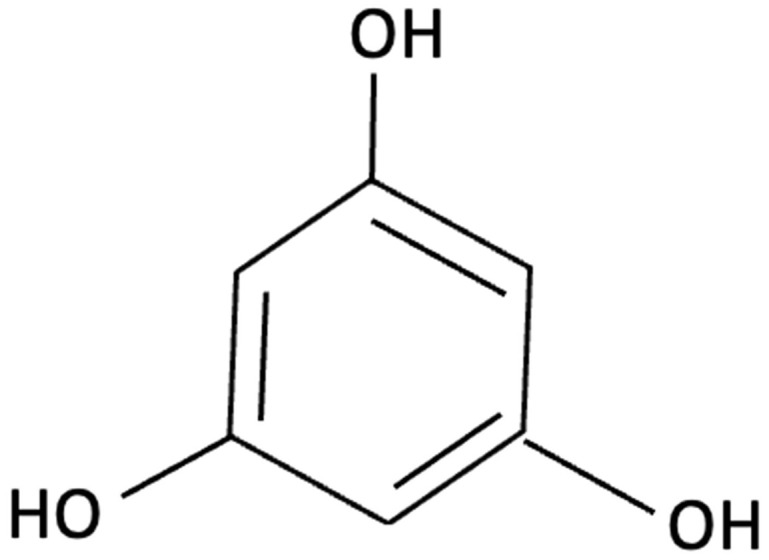	Brown Seaweed	Neuroprotective	*In vitro* assays using HT-22 and primary hippocampal neurons were conducted against Aβ1–42-induced cytotoxicity, oxidative stress, and synaptic damage, supported by *in vivo* stereotaxic hippocampal injection and behavioural testing in 5XFAD mice.	Phloroglucinol significantly reduced Aβ-induced ROS accumulation and synaptic loss in vitro and improved spatial learning and working memory in 5XFAD mice.	[157]
PFF-A (Phlorotannin-s)	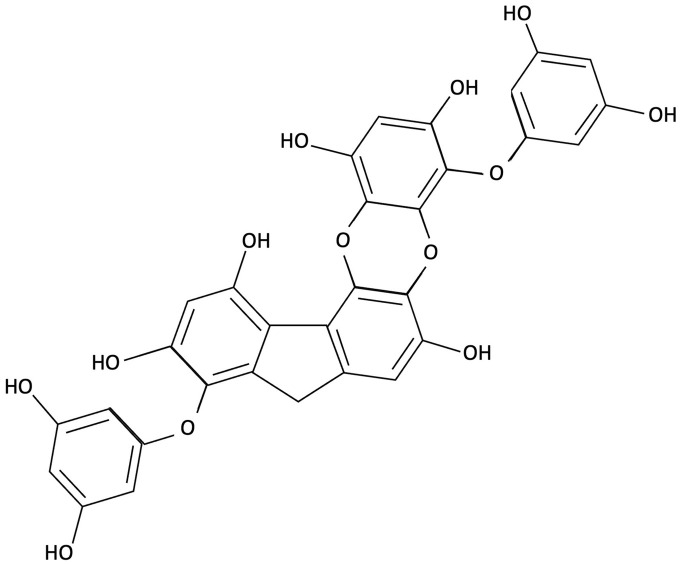	Ecklonia cava, Brown Seaweed	AChE inhibition, Neurotransmitter modulation	Male ICR mice received oral PFF (0.2 or 2 mg/kg/day) for 7 days. Memory was evaluated using passive avoidance; neurotransmitter analysis and AChE inhibition were also conducted.	Improved cognitive performance in memory-impaired mice, normalised hippocampal norepinephrine and glutamate, decreased GABA, increased 5-HT and Ach and inhibited AChE (IC_50_ ≈ 27.4 µM).	[148]
Saringosterol	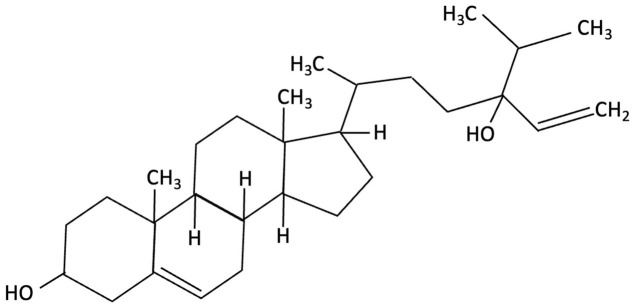	Sargassum fusiforme, Brown Seaweed	Anti-inflammatory	Male APPswePS1ΔE9 and WT mice received daily oral gavage of 24(S)-saringosterol (0.5 mg/25 g) for 10 weeks to assess cognitive effects.	Significantly prevented cognitive decline in APPswePS1ΔE9 mice, improving spatial and object memory (OLT and ORT), likely via LXR-mediated microglial modulation.	[158]
k-carrageenan	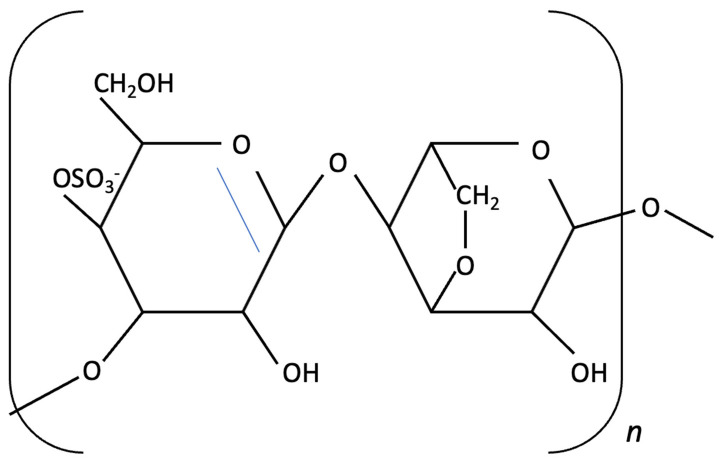	Kappaphycus alvarezii, Red Algae	Anti-inflammatory, Neuroprotective	*In vitro* LPS stimulated neuroinflammation in Murine microglial N9 cell. Treated with KOS or desulphated derivatives (DSK)	Attenuated neuroinflammation by reducing NO, TNF-, and IL-10 release, inhibited microglial over proliferation and preserved resting microglial morphology.	[159]
Lutein and Zeaxanthin	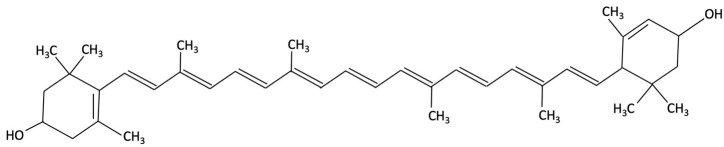 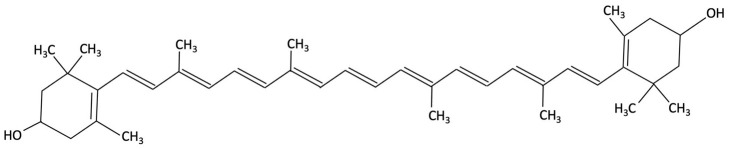	Porphyra (Nori), Red Seaweed	Neuroprotective	6-month randomised, double-blind, placebo-controlled trial in adults (40–75 years) assessing daily supplementation of lutein (10 mg) and zeaxanthin (2 mg).	Improvements in visual episodic memory compared to placebo and visual learning.	[160]
Astaxanthin (Ax-Hp)	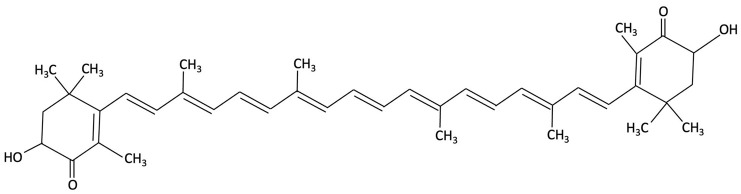	Haematococcus pluvialis, Green Algae	Antioxidant, Anti-inflammatory	A 12-week randomised, double-blind, placebo-controlled trial in healthy middle-aged adults (aged 45–64 years) with age-related subjective cognitive complaints.	High-dose supplementation improved one CogHealth task, response time and accuracy, with trends in three others. GMLT total errors significantly decreased by week 4, suggesting cognitive benefits.	[161]
Astaxanthin (Ax-Hp)	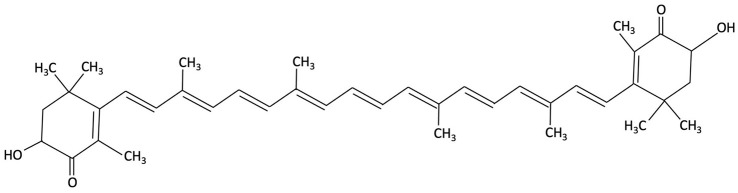	Haematococcus pluvialis, Green Algae	Antioxidant	12-week randomised, double-blind, placebo-controlled trial (*n* = 30) healthy adults (50–69 years), receiving 0 mg, 6 mg or 12 mg/day.	Significantly increased erythrocyte Ax-Hp levels and reduced PLOOH, indicating antioxidant activity.	[162]
Macular Xanthophylls		Chlorella and Dunaliella species, Green Algae	Antioxidant, Anti-inflammatory, Neurotrophic Modulation	6-month randomised, double-blind, placebo-controlled trial in healthy young adults (18–25 years), assessing dose-dependent effects of macular xanthophyll supplementation (13 mg or 27 mg/day) on cognitive and biochemical outcomes.	Supplementation improved composite and verbal memory, attention and processing speeds. BDNF, AOC, MPOD, lutein and zeaxanthin increased; IL-1β decreased. Cognitive gains correlated with BDNF and MPOD changes.	[163]
Ulvan	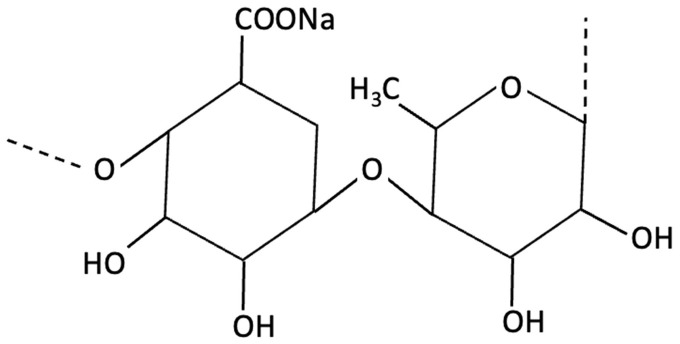	Ulva lactuca, Green Seaweed	Neuroprotective	*In vitro* study using SH-SY5Y neuroblastoma cells of Ulva against BPA-induced toxicity.	Strong antioxidant, anticholinesterase and neuroprotective effects by restoring cell viability, inhibiting capase-3 activation.	[164]
Mixed Compounds, not explicitly recorded		Mixed Edible Algae	Not directly assessed	Cross-sectional analysis of 2018 CLHLS data from older Chinese adults (≥65 years), examining associations between edible mushroom/algae intake and cognitive impairment.	In the fully adjusted model, cognitive impairment risk was reduced by 29% (OR: 0.710) for daily intake and 25.3% (OR: 0.747) for occasional intake.	[165]

The arrow represents a decrease level. Abbreviations: AOC: Antioxidant capacity, COX-2: cyclooxygenase-2, GFAP: glial fibrillary acidic protein, GABA: gamma-aminobutyric acid, GMLT: Groton maze learning test, Iba-1: allograft inflammatory factor 1, ICV: intracerebral ventricular, IL: interleukin, iNOS: inducible nitric oxide synthase, IOE: *I. Okamurae* extracts, JNK: c-Jun N-terminus kinase, KOS: κ-carrageenan oligosaccharides, LPS: lipopolysaccharide, LXR: liver X receptor, MAPK: mitogen-activated protein kinase, MPOD: macular pigment optic density, OLT: object location test, ORT: object recognition test, PARP: poly (ADP-ribose) polymerase, PLOOH: phospholipid hydroperoxide, PT: *Phaeodactylum tricornutum*, 4-HNE: 4-hydroxynoneal, 5-HT: serotonin.

## Data Availability

No new data were created or analysed in this study.

## Supplementary Materials

The following supporting information can be downloaded at: https://www.mdpi.com/article/10.3390/molecules30224456/s1.

## Abbreviations

The following abbreviations are used in this manuscript:

Aβ	Amyloid-beta
ACh	Acetylcholine
AChE	Acetylcholinesterase
AChEIs	Acetylcholinesterase inhibitors
ADAM10	a disintegrin and metalloproteinase domain-containing protein 10
AICD	APP intracellular domain
AMPA	α-amino-3-hydroxy-5-methyl-4-isoxazolepropionic acid
AOC	Antioxidant capacity
APP	Amyloid precursor protein
ARIAs	amyloid-related imaging abnormalities
ARIA-E	vasogenic oedema
BACE1	β-site APP-cleaving enzyme 1
BDNF	brain-derived neurotrophic factor
COX-2	cyclooxygenase-2
CREB	Cyclic AMP-responsive element-binding protein
CTNF	ciliary neurotrophic factor
DLPFC	dorsolateral prefrontal cortex
EGCG	epigallocatechin gallate
GFAP	Glial fibrillary acidic protein
GABA	Gamma-aminobutyric acid
GMLT	Groton maze learning test
GSK3β	Glycogen synthase kinase-3 beta
Iba-1	allograft inflammatory factor 1
ICV	intracerebral ventricular
IL	interleukin
iNOS	inducible nitric oxide synthase
IOE	*I. Okamurae* extracts
JNK	c-Jun N-terminus kinase
KOS	κ-carrageenan oligosaccharides
LPS	lipopolysaccharide
LTP	long-term potentiation
LXR	liver X receptor
MAB	monoclonal antibodies
MAPK	mitogen-activated protein kinase
MAPK/ERK	mitogen-activated protein kinase/extracellular signal-regulated kinase
MCI	Mild cognitive impairment
MDA	malondialdehyde
MPOD	macular pigment optic density
mtDNA	Mitochondrial DNA
NF-κB	nuclear factor kappa-light-chain-enhance of activated B cells
NFTs	Neurofibrillary tangles
NO	nitric oxide
OLGs	oligodendrocyte lineage cells
OLT	object location test
OPCs	oligodendrocyte precursor cells
ORT	object recognition test
PARP	poly (ADP-ribose) polymerase
PI3K/Akt	phosphatidylinositol 3-kinase/protein kinase B
PLOOH	phospholipid hydroperoxide
PT	*Phaeodactylum tricornutum*
P38-MAPK	P38 mitogen-activated protein kinase
RCF	Respiratory chain function
RCTs	Randomised controlled trials
ROS	Reactive oxygen species
sAPP	Soluble
TNF	tumour necrosis factor
4-HNE	4-hydroxynoneal
5-HT	serotonin
6-OHDA	6-hydroxydopamine
α7 nAChRs	α7 subtype of nicotinic acetylcholine receptors
