# Supplementary material for: Therapeutic Potentials of the Seaweed-Derived Compounds for Alzheimer’s Disease
Source: Molecules. 2025 Nov 19;30(22):4456. doi: 10.3390/molecules30224456 (PMC12655838; doi:10.3390/molecules30224456)
Supplement: Supplementary file 1 [file molecules-30-04456-s001.zip › molecules-3918267-supplementary.pdf]

Supplementary material

Therapeutic Potentials of the Seaweed-Derived Compounds for Alzheimer's Disease

Keanie Ward [1], Michael H. Cole [1,2], Lyn R. Griffiths [3], Heidi G. Sutherland [3], Pia Winberg [4,5], Barbara J. Meyer [5,6] and Francesca Fernandez [1,2,3,*]

[1] School of Behavioural and Health Sciences, Faculty of Health Sciences, Australian Catholic University, Banyo, QLD 4014, Australia; keanie.ward@myacu.edu.au (K.W.); michael.cole@acu.edu.au (M.H.C.)
[2] Healthy Brain and Mind Research Centre, Australian Catholic University, Fitzroy, VIC 3065, Australia
[3] Centre for Genomics and Personalised Health, School of Biomedical Sciences, Queensland University of Technology, 60 Musk Ave, Kelvin Grove, QLD 4059, Australia; lyn.griffiths@qut.edu.au (L.R.G.); heidi.sutherland@qut.edu.au (H.G.S.)
[4] Venus Shell Systems Pty Ltd., Huskisson, NSW 2540, Australia; pia@venusshellsystems.com.au
[5] School of Medical, Indigenous and Health Science, University of Wollongong, Wollongong, NSW 2522, Australia; bmeyer@uow.edu.au
[6] Molecular Horizons, University of Wollongong, Wollongong, NSW 2522, Australia
* Correspondence: francesca.fernandez@acu.edu.au

Supplementary Methodology

Search methodology literature search was conducted across multiple electronic databases, including Google Scholar and Pubmed (Medline) to identify studies investigating the neuroprotective potential of seaweed-derived bioactive compounds in the context of AD and cognitive decline. Search terms utilised keywords related to seaweed (e.g., "seaweed", "macroalgae", "marine algae", "brown algae", "red algae" and "green algae") and terms relevant to neurological and cognitive outcomes (e.g., "Alzheimer*", "cognitive decline", "neuroprotect*", "oxidative stress", "neuroinflammation" and "tau"). Later searches were then employed after identifying specific compounds in the initial search, which observed the specific bioactive compounds entered into the search (e.g., "fucoidan", "dieckol", "ulvan", "phloroglucinol", "diphlorethohydroxycarmalol", "fucoxanthin", "lycopene", "fucosterol", "saringosterol", "carrageenan", "lutein", "zeaxanthin", "astaxanthin", "macular xantho-phylls"). Boolean operators "AND" and "OR" were used to combine the related concepts, and truncation (*) was applied to capture variations in terminology, seen throughout the initial search. After abstract screening, only full text peer-reviewed articles were included. Both preclinical and clinical evidence were included to capture molecular, mechanistic and translational findings. Exclusion criteria included studies published in English. No restrictions were placed on the year of publication to ensure that both foundational research and recent developments were captured.
